# Consensus standards for the process of cancer care: a modified expert panel method applied to head and neck cancer. South and West Expert Tumour Panel for Head and Neck Cancer.

**DOI:** 10.1038/bjc.1998.319

**Published:** 1998-06

**Authors:** M. A. Birchall

**Affiliations:** University Department of Otolaryngology-Head & Neck Surgery, Southmead Hospital, Bristol, UK.

## Abstract

There are many pressures to improve the standard of care delivered to cancer patients, including the reforms subsequent to the Calman-Hine report. The establishment of standards is a prerequisite for audit, benchmarking and certification of cancer centres and units. Randomized trials of head and neck cancer are uncommon, and other forms of evidence often conflicting. In the south and west of England, a multidisciplinary expert panel consensus method has been applied to the development of standards. A panel representative of specialties involved in the process of care at all three levels, plus social medicine and lay members, was constructed. A model for the process of care was developed consisting of activity areas. For each activity, a near exhaustive list of tasks and standards was established. A three-iteration method with statistical group response was then used to refine the standards. The same method was also applied to the production of a minimum data set for registration, recording and audit. The resulting standards will be regularly reviewed. We have developed a model of the care process, and an expert panel methodology that is applicable to a wide range of problems in clinical oncology.


British Journal of Cancer (1998) 77(11), 1926–1931
© 1998 Cancer Research Campaign

### Consensus standards for the process of cancer care: a modified expert panel method applied to head and neck cancer

**MA Birchall[1] and the South and West Expert Tumour Panel for Head and Neck Cancer**

[1]University Department of Otolaryngology – Head & Neck Surgery, Southmead Hospital, Bristol, BS10 5ND, UK

**Summary** There are many pressures to improve the standard of care delivered to cancer patients, including the reforms subsequent to the Calman–Hine report. The establishment of standards is a prerequisite for audit, benchmarking and certification of cancer centres and units. Randomized trials of head and neck cancer are uncommon, and other forms of evidence often conflicting. In the south and west of England, a multidisciplinary expert panel consensus method has been applied to the development of standards. A panel representative of specialties involved in the process of care at all three levels, plus social medicine and lay members, was constructed. A model for the process of care was developed consisting of activity areas. For each activity, a near exhaustive list of tasks and standards was established. A three-iteration method with statistical group response was then used to refine the standards. The same method was also applied to the production of a minimum data set for registration, recording and audit. The resulting standards will be regularly reviewed. We have developed a model of the care process, and an expert panel methodology that is applicable to a wide range of problems in clinical oncology.

**Keywords:** standards; process; consensus method; head and neck cancer

Head and neck cancer makes up about 6% of new cancers in the south and west of England (South and West Cancer Intelligence Unit data) and is an increasing problem in most parts of the world (Gile et al, 1994; Tobias, 1994). It is a heterogeneous disease, with its behaviour being histology and site dependent (Maran et al, 1993). The functional and psychological effects of head and neck cancer are as profound as those seen in any other form of malignancy (Lansky et al, 1988). Yet, despite advances in radiotherapy and surgery, including increased interdisciplinary co-operation, 5-year mortality rates have not improved for 30 years (Stell 1992; Gile et al, 1994). These problems are not unique to head and neck cancer, however, and the study of how patients progress from first symptoms to eventual outcomes represents an important model for the study of care processes in clinical oncology. The results of experience and experiment using this model should be transferable to other cancer sites.

A standard is a 'quality or specification by which something may be tested or measured' (*Oxford English Dictionary*). It is 'a precise and authoritative statement of the criteria necessary to ensure that a process is fit for the purpose for which it is intended'. To effectively facilitate improvements in care, standards have to meet some important criteria (Dale and Oakland, 1991). They must be practical, prepared in response to a recognized need, evidence based, regularly reviewed and prepared at a broad level.

There is good evidence for the need for standards in head and neck cancer care. Studies have indicated considerable disparity in treatments for patients with head and neck cancer (Maher and Jefferis, 1990), and that management decisions depend on the background of the clinician responsible for care (O'Sullivan et al, 1994) and the geographical location of the patient (O'Sullivan et al, 1994; Bradley, 1989). In paediatric oncology, in which standards have been in place for some time, survival rates have increased (Stiller, 1988).

The report of the Expert Advisory Group on Cancer, the 'Calman–Hine Report' (Calman and Hine, 1995), suggests guidelines for early and urgent referral as well as improvements in feedback to GPs. Site-specific statements of intent on these issues were supplied by all hospitals as part of the application process for provisional cancer centre or unit status. Now that cancer centres and units have been nominated, there is the potential for a proliferation of 'guidelines' by the many hospitals involved. This makes the need for valid regional standards an urgent one.

As a result of the implementation of this report, it has been made a prerequisite of the conferment of cancer centre status that standards exist and be used. A further requirement is the reinforcement of health authority and professional body requirements for audit, and effective audit also requires established standards.

The financial cost of treating patients with head and neck cancer (Million, 1994) is high. In the absence of clear standards, providers of care at all levels may well adopt cheaper options for care rather than those that are likely to provide better outcomes for patients. For all these reasons, therefore, there is a very clear need to be able to measure the standard of head and neck care at all levels, from primary care upwards.

The requirement of an evidence base for standards presents particular difficulties in the current setting. In common with other malignancies of intermediate incidence, most of the evidence base in head and neck oncology is non-experimental in nature, with relatively few reported randomized controlled clinical trials of sufficient study power (Kelly, 1997). There remains considerable dispute about the correct therapy for many sites and stages of

*Received 26 June 1997*
*Revised 25 November 1997*
*Accepted 27 November 1997*

*Correspondence to:* MA Birchall disease, such as $T_3$ carcinoma of the larynx. Poor registration of cancers has made extrapolation from these data sources difficult. For example, a recent study of head and neck cancer in the south west revealed that less than 2% of cases had stage data recorded (Thorne et al, 1997). These factors present considerable difficulties for those attempting to establish standards and guidelines for care. Fortunately, despite these problems, the use of qualitative, consensus methodology may still allow meaningful standards to be developed based on experience and 'expert opinion' (Jones and Hunter, 1996). A review of the available consensus methods suggested that the expert panel, or nominal group, method might be the most appropriate.

The aims of the current study are to develop a model for the process of head and neck cancer care, to determine the applicability of a modified expert panel technique in determining standards for this area of oncology, to use this technique to obtain a set of regionally agreed standards for head and neck cancer care and to establish a minimum data set for head and neck cancer for recording, audit, research and registration purposes.

### METHODS

The method selected for establishing standards for head and neck cancer care was a modified expert panel (or nominal group) method. This is a consensus method, originally developed in the USA, and already has been usefully applied to health service problems. For example, the derivation of appropriate outcome measures in orthopaedics (Liang et al, 1991). Like other consensus methods, this technique allows synthesis of standards when little and/or contradictory information exists, as is the case for head and neck cancer.

### Assembling the panel

Requirements for assembling the panel were that composition should be representative of those professionals involved in care of patients with head and neck cancer, and that each member could, in some way, be regarded as having expertise in this area (Jones and Hunter, 1996). In addition, members were also selected to ensure representation of three levels: primary care, district general hospital and teaching hospital. These approximate to the levels subsequently identified by the Calman–Hine Report (Calman and Hine, 1995; Haward, 1995): primary care, cancer 'units' and cancer 'centres'. Composition according to the two methods of classification is shown in Figure 1. As the discussions included many topics of general interest with implications for service configurations, representatives from public health medicine were also included. Finally, membership was checked and refined to ensure a broad geographical representation across the south and west. It has been shown previously that doctors selected in this way express views that are representative of their colleagues (McKee et al, 1991).

### Protocol

The process used to determine standards for head and neck cancer care is shown in Figure 2. At the first meeting, the aims and objectives were outlined and a chairperson was elected. An outline pathway for the process of head and neck cancer was sketched out by the use of a 'brainstorming' technique, and then activity areas at various points along the process were allocated to individual panel members for development.

**Figure 1** Composition of the south and west expert panel for head and neck cancer by specialty and Calman–Hine level. Pie charts showing the composition of the expert panel by 'level' of care. (**A**) pre-Calman–Hine; (**B**) post-Calman–Hine

**Figure 2** Modified expert panel method used for preparation of standards for the process of head and neck cancer care. Flow-chart showing the expert panel method used for preparation of standards for the process of head and neck cancer care

© Cancer Research Campaign 1998

**Figure 3** Model of the process of head and neck cancer care developed for this study in patients presenting for the first time. Each box represents a discrete activity area

At the following three meetings, panel members presented an analysis of their views on the main points within their activity area, illustrated with reference to published evidence whenever possible. Each activity area in turn was discussed and a near exhaustive set of provisional standards determined (first iteration). The same process was applied to determination of a minimum data set.

When all presentations were completed, the standards were tabulated. These tables were distributed to each panel member. Panel members were asked to record their views on each standard in an open-ended manner. They were also asked to grade the importance of each standard for the process of head and neck cancer care on a 0–10 linear analogue scale. Replies were collected and open-ended comments collated. Anonymity of responses was maintained. The same method was applied to points within a minimum data set. Linear analogue responses were presented as summary statistics (median and range).

The results of the anonymous survey were re-presented to the panel, and further discussion and refinement completed (second iteration). Those points scoring low median marks were addressed in turn, allowing the reduction of the original standards list by 11 points, and the original data set by 5 points.

At this stage, the results were again collated and recirculated to check for inconsistencies (third iteration) before finalizing. One inconsistency was identified and rectified at this stage.

### RESULTS

The first phase of the study required the development of a model for the process of head and neck cancer care. This is shown in Figure 3. Each box represents a separate activity area for standards, each of which is divided into a series of tasks. The hierarchical model for this breakdown is shown in Figure 4. An example of the standards for a particular activity area is represented in Table 1. The intervals between each activity were the subject of a separate series of minimum standards, shown in Table 2. The minimum data set determined by the expert panel method are shown in Table 3.

**Figure 4** Diagram demonstrating the hierarchical structure adopted for defining standards

© Cancer Research Campaign 1998

**Table 1** An exert from the standards for the process of head and neck cancer care developed by the present study

| Activity | Task | Standard |
|---|---|---|
| Diagnosis and staging | Responsibility | 95% should be by a head and neck specialist<br>TNM staging in 100% |
| | Examination under anaesthesia (EUA) | 50% of oral cavity carcinoma<br>90% of other tumours |
| | Radiology | 90% of radiological investigations should be performed and reported before treatment planning<br>CT/MRI 90% T3/T4 tumours at all sites and 100% of nose/sinus/ear tumours<br>CXR 100% of all head and neck cancers<br>OPG 100% oral and oropharyngeal tumours |
| | Biopsy | 100% of new cases require a histological diagnosis of cancer prior to treatment planning |
| | Fine-needle aspiration biopsy | Cytological examination of fine-needle aspirates should be available in 95% of units and centres<br>80% of neck masses<br>80% of parotids |

For definitions of process, activity and tasks see Figure 4.

**Table 2** Maximum acceptable intervals between activities in the process of care

| Time between activities | Standard |
|---|---|
| First symptoms to GP/GDP presentation | 1 month |
| GP/GDP to first outpatients | 10 working days |
| Clinic note to GP/GDP | 5 working days |
| Fine-needle aspiration biopsy/biopsy arrival at pathology department | no wait |
| Time for frozen section result | 30 min for one, 45 min for multiple |
| Biopsy to report issue | 5 working days |
| General clinic to specialist H&N OPD | 10 working days |
| H and N OPD to EUA/panendoscopy/dental/prosthetic | 5 working days |
| H and N OPD to radiotherapy, chemotherapy (curative intent) | 10 working days to planning for RTX or start CTX |
| H and N OPD to radiotherapy, chemotherapy (palliative intent) | 5 working days to planning |
| H&N OPD to ablative surgery | 10 working days |
| Within radiotherapy or chemotherapy course | As planned and documented |
| Primary treatment to rehabilitation (speech, swallowing, needs assessment) | No delay |
| Treatment to first follow-up clinic | 1 month |

GP, general practitioner; GDP, general dental practitioner; H and N, head and neck; OPD, outpatient department; EUA, examination under anaesthetic.

### DISCUSSION

The determination of standards is essential for site-specific areas of oncology. Here, a modified expert panel method has been applied to the process of head and neck cancer care in the south and west of England. The result has been the distillation of standards for the process of care, for times between activities and a minimum data set for recording, registration and audit purposes.

There have been previous attempts to assemble quality assurance documents for head and neck cancer in the UK (Glaholm et al, 1995; Rhys-Evans, 1995). These documents represent guidelines for care, rather than true standards, and have not used formal consensus methods in their construction. Standards including defined (preferably numerical) targets to be reached in order to be associated with the minimum level consistent with a high standard of care are uncommon. The current study shows that, given sufficient time and thought, it is possible to use a representative sample of experts to produce standards with set targets across a wide range of activity areas within head and neck cancer care.

It is necessary for us to look again at the requirements for standards, and to examine how well the results of the expert panel method fulfilled them. The need for standards is clear, as described above. Wherever possible, the standards were drawn up with reference to the evidence base alluded to above, but for the majority of areas sufficient disagreement existed to make consensus evidence the only valid option. Nonetheless, it is acknowledged that there are ongoing trials in this field, the results of which will require regular review under the current standards (UKCCR, 1991; Dische, 1995), for standards can only be as good as the evidence available at the time they were drawn up. It is currently planned to

© Cancer Research Campaign 1998 

**Table 3** Minimum data set for head and neck cancer determined by the present study

| Area | Point |
|---|---|
| Patient identifiers | Hospital<br>GP/GDP<br>Name<br>Date of birth<br>Hospital number |
| Co-morbidity | ASA at first combined clinic (ASA, 1987)<br>QOL at presentation (EORTC, 1995)<br>QOL at 6/12 follow-up |
| Pathology | Tumour site/subsite (ICD10, 1994)<br>Is this a recurrence (yes/no)<br>Clear margins (yes/no)<br>Number positive nodes<br>Extracapsular spread (yes/no)<br>Perineural spread (yes/no)<br>Perivascular spread (yes/no)<br>Histological type |
| Radiology | CXR, OPG, CT, MRI (whether performed yes/no) |
| Staging | Clinical stage (TNM, AJCC, 1988)<br>Clinical stage (EUA)<br>Radiological stage (CT, MRI)<br>Pathological stage |
| Process | Dates of each event in disease/treatment |
| Surgery | Operation type<br>Named consultant surgeon (RCS coded)<br>Reconstruction method<br>Named reconstructor (RCS coded)<br>Immediate complications (first 24 hours)<br>Early complications (less than 30 days)<br>Late complications (more than 30 days) |
| Radiotherapy | Named consultant radiotherapist and oncologist<br>Radiotherapy intent: curative/palliative<br>Radiotherapy type<br>Dose, fractionation and duration (actual)<br>Gaps other than weekends (yes/no)<br>Response of tumour (CR or <CR)<br>Early complications<br>Late complications |
| Chemotherapy | Chemotherapy type<br>Complications<br>Response of tumour (CR, PR, nil)<br>Death location<br>Head and neck cancer given as a cause of death (yes/no) |

review the standards every 2 years, unless major breakthroughs in treatment, such as those suggested by work on gene therapy, occur in the interim. If standards are not so reviewed, they may become irrelevant or, worse, may actually hinder progress by suggesting outdated goals.

Standards should be prepared to the broadest level consistent with meeting the needs of all parties concerned within a reasonable time-scale. In the case of a multidisciplinary process, such as head and neck cancer care, we would argue that the minimum acceptable level is regional. However, some would argue that if the need for standards exists, the correct level would be either national or international (e.g. European). Such standards might be accepted and used more readily than those produced at a more local level. Yet, the preparation and, crucially, the long-term maintenance of meaningful standards, especially if formal consensus methods are used, is time consuming and potentially costly. Certainly, in the UK or the EU there is no central funding for standard setting in oncology. Until such funding exists, meaningful initiatives at a supra-regional level may remain impractical.

The completion of this study has taken more than 2000 man hours of expert panel member time. So far, this time has been given freely by those involved, without any sessional payments or centrally funded travel costs. If repeated regularly and for all site-specialist areas, this exercise would have considerable service and cost implications. As the need for standards is no less compelling in any other area of oncology, we would argue that there is a need for both central funding and official recognition of standards produced in a systematic and representative manner.

The regular revisiting of standards requires considerable discipline on the part of an expert panel. In practice, there is no reason why the membership of the panel should not change if members felt they had contributed sufficiently. In fact, such turnover of membership would form a valuable part of the continuous improvement process, preserving representativeness as service configurations change with time.

Standards are, of necessity, provisional. It has been suggested that the results of consensus methods may represent 'collective ignorance' just as much as wisdom (Jones and Hunter, 1996). As a result, it is important to validate them by reference to actual practice. The standards, although distilled, are still too wide ranging to be validated by a single audit. However, a prospective audit has been set up, based on elements of the standards, to examine the 'cancer journey' for patients with head and neck cancer. Other points will be tested by local audits, and a few by the results of ongoing randomized clinical trials (UKCCR, 1991; Dische, 1995). Standards also have to be practical, and the panel noted that part of this practicality is financial. As the Calman–Hine reforms are intended to be 'cost neutral', money may prove to be the biggest limiting factor of all.

The selection of the panel was made to ensure fair representation in terms of geography, specialty and size of institution. At the time the panel was originally constituted, trusts in the south and west were divided into teaching hospitals and district general hospitals, and equal numbers of members drawn from each. Subsequently, trusts in the region have been designated as cancer centres or units in such a way that the balance of the panel may seem skewed (Figure 1). Nevertheless, we feel that the membership remains representative of those currently involved in head and neck cancer care in the south and west.

In addition to the prerequisites for standards outlined above, the Department of Health in its 'guidelines for guidelines' has added the requirement of taking into account 'patient choices and values' (NHSME, 1993). To this, we could reasonably add, as do Calman and Hine (1995), the choices and values of carers. It is easy to see why previous attempts at quality documents for head and neck cancer (Glaholm et al, 1995; Rhys-Evans, 1995) avoided this aspect as the assaying of such choices and values with any degree of validity requires time- and resource-consuming methods such as interviews and focus groups (Kitzinger, 1995). As an illustration, those patient and patient group representatives initially invited onto the panel felt too inhibited by the process to contribute fully. Further, the representativeness of one or two such persons could be seriously questioned. Therefore, it is an important future phase of development that these views be sought with particular reference to the current results, leading to further refinement.

© Cancer Research Campaign 1998

Another objection to the use of standards in clinical practice is the perceived potential for 'abuse' by health authorities and trusts and patient groups, particularly in the area of litigation. However, we would argue that the adoption of set standards empowers units and centres to press for greater resources to achieve them, whereas achievement of established standards provides a powerful defence against potential litigation. Viewed in these ways, standards are clearly the clinicians' friend.

The establishment of a minimum data set for a site-specific area of oncology is an important step that allows standardized recording for head and neck cancer, and facilitates audit, research and registration. An agreed minimum data set for site-specific cancers is also necessary for the establishment of an effective computerized database. Such databases are a further prerequisite for the granting of cancer centre or unit status to provider trusts. We found the expert panel method particularly applicable to this part of the study as the data set is a simple list that is easily refined by the statistical feedback technique used.

The establishment of standards is a crucial early step on the road to planned, steady improvement in cancer care. We have applied a modified expert panel consensus technique to the distillation of standards for care in head and neck cancer. The standards produced represent a comprehensive tool for internal improvement and comparative studies, including audit. In addition, we have produced a minimum data set to assist in the standardization of recording of cases for audit, research and registration purposes. Further work is required to validate and refine the standards by 'field-testing', research and the incorporation of patient and carer views. The standards will be reviewed biannually to maintain relevance. The modified expert panel technique is appropriate for the distillation of site-specific standards for care in clinical oncology.

### ACKNOWLEDGEMENTS

The South and West Expert Tumour Panel for Head and Neck Cancer comprises the following, all of whom have freely given many complete days to this project: Chris Baughan, Oncology, Southampton; John Boyles, purchasing representative, Gloucestershire; Mike Bridger, ENT, Plymouth; Perric Crellin, Oncology, Poole; John Eveson, Pathology, Bristol; Tim Flood, Maxillofacial, Salisbury; Karen Forbes, Palliative Care, Bristol; Phil Guest, Maxillofacial, Bristol; Kay Howe, Regional Cancer Organisation; Julian Kabala, Radiology, Bristol; Liz Lee, GP, Bristol; Pat MacCleod; Hugh Newman, Oncology, Bristol; Chris Randall, ENT, Southampton; Peter Saxby, Plastic Surgery, Exeter; Jenifer Smith, Cancer Intelligence Unit, Winchester; John Waldron, ENT, Bath; Graham Zaki, Maxillofacial, Portsmouth. We would all like to thank Dr Archie Prentice, Plymouth and Dr Neil Cowley, Poole, whose ideas initiated this study. The practical running of the Tumour Panel has been made possible by Dr Kaye Howe and the staff at the Regional Cancer Organisation. Tireless secretarial support has been provided by Eva Hicks of the Department of Otolaryngology, Southmead Hospital, Bristol. This work has been funded in part by the Wessex Cancer Trust and the South West Regional Health Authorities.

### REFERENCES

Bradley PJ (1989) Survey of current management of laryngeal and hypopharyngeal cancer. *J R Coll Surg Edinb* **34**: 197–200

Calman KC and Hine D Expert Advisory Group on Cancer (1995) *A Policy Framework for Commissioning Cancer Services: a Report to the Chief Medical Officers of England and Wales*. DoH and the Welsh Office: London

Dale B and Oakland J (1991) *Quality Improvement Through Standards*. Stanley Thomas: Cheltenham

Dische S (1995) EORTC trial of continuous hyperfractionated radiation therapy: interim results. *Presented at the Annual Meeting of the British Oncological Association, March 1998*.York

Gile G, Thursfield V and Staples M (1994) The bottom line: trends in cancer mortality, Australia 1950–1991. *Cancer Forum* **18**: 12–23

Glaholm J, Brown A and Das Gupta A (1995) Provision and quality assurance for head and neck cancer care in the United Kingdom: a nationally co-ordinated multidisciplinary approach. *The British Association of Head and Neck Oncologists, consultation document*

Haward RA (1995) Establishing cancer units. *Br J Cancer* **72**: 531–534

Jones J and Hunter D (1996) Consensus methods for medical and health services research. In *Qualitative Research in Health Care*, Mays N and Pope C (eds), pp. 46–58. BMJ Publishing Group: London

Kelly GA (1998) Retrospective study into evidence-based medicine in otolaryngology. *Clin Otolaryngol* (in press.)

Kitzinger J (1995) Introducing focus groups. *Br Med J* **311**: 299–302

Lansky SB, List MA, Ritter-Sterr C, Logemann J and Willis M (1988) Performance parameters in head and neck cancer patients. *Proc Annu Meet Am Soc Clin Oncol* **7**: A603

Liang MH, Katz JN, Phillips C, Sledge C and Cats-Baril W (1991) The total hip arthroplasty outcome evaluation form of the American Academy of Orthopaedic Surgeons. Results of a nominal group process. The American Academy of Orthopaedic Surgeons Task Force on Outcome Studies. *J Bone Joint Surg* **773**: 639–646

McKee M, Priest P, Ginzler M and Black N (1991) How representative are members of expert panels? *Qual Assur Health Care* **3**: 89–94

Maher EJ and Jefferis AF (1990) Decision making in advanced cancer of the head and neck: Variation in the views of medical specialists *J R Soc Med* **83**: 356–359

Maran AG, Wilson JA and Gaze M (1993) *Stell and Maran's Head and Neck Surgery*, 3rd edn. pp. 1–6. Butterworth Heinemann: Oxford

Million RM and Sigal MC (1994) Cost of management of head and neck cancer. In *Management of head and neck cancer: a multidisciplinary approach*, 2nd edn. Million RM and Cassisi NJ (eds). JB Lippincott: Philadelphia

NHSME (1993) *Improving Clinical Effectiveness*. Document EL(93)115. Department of Health: London

O'Sullivan B, Mackillop W, Gilbert R, et al (1994) Controversies in the management of laryngeal cancer: results of an international survey of patterns of care. *Radiother Oncology* **31**: 23–32

Rhys-Evans PH (1995) Provision and quality assurance for head and neck cancer care in the United Kingdom: a nationally co-ordinated approach. *The Royal Marsden Hospital*, March

Stell PM (1992) Adjuvant chemotherapy in head and neck cancer. *Semin Radiat Oncol* **2**: 195–205

Stiller CA (1988) Centralisation of treatment and survival rates for cancer. *Arch Dis Child* **63**: 23–30

Tobias JS (1994) Current issues in cancer: cancer of the head and neck. *Br Med J* **308**: 961–966

Thorne P, Etherington D and Birchall MA (1997) The effect of social class on head and neck cancer in the South West of England: a population based study. *Clin Otolaryngol* (in press)

UKCCCR Head and Neck Collaborative Group (1991) UKHAN1: a trial of chemotherapy with radiotherapy in the treatment of advanced squamous carcinoma of the head and neck. CRC Trials Group: London

© Cancer Research Campaign 1998


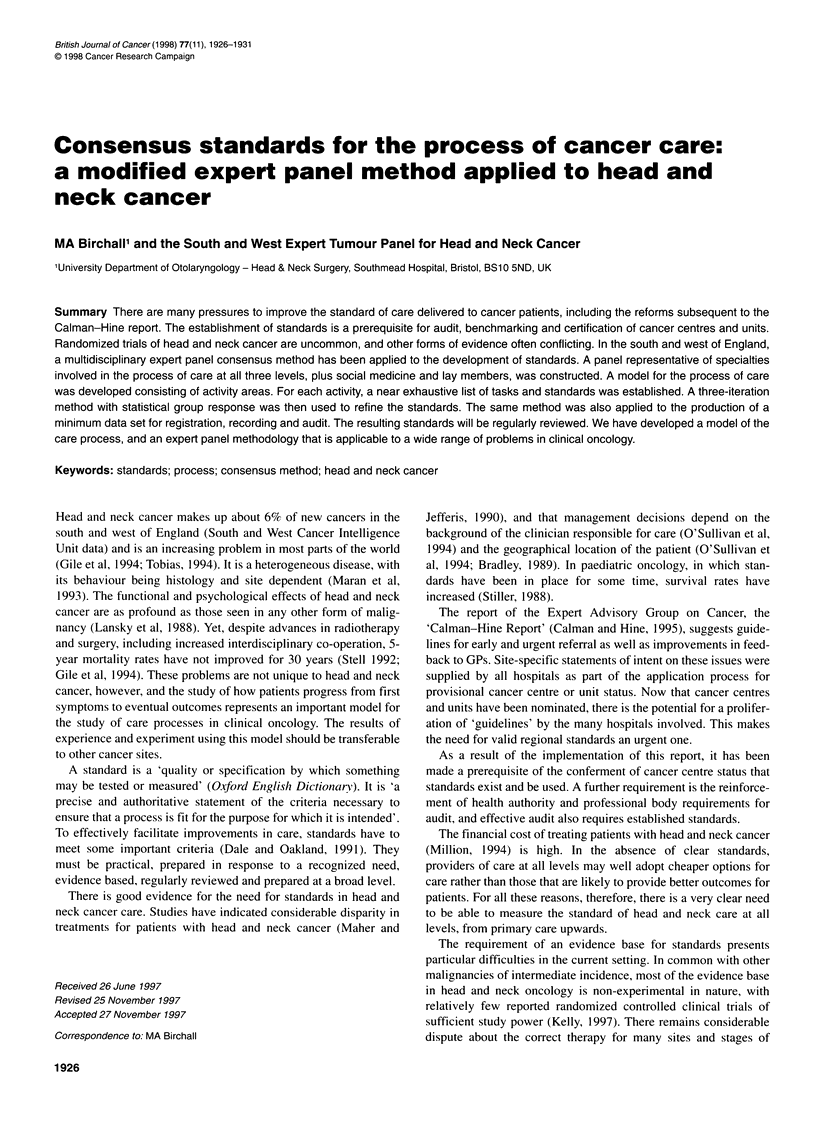

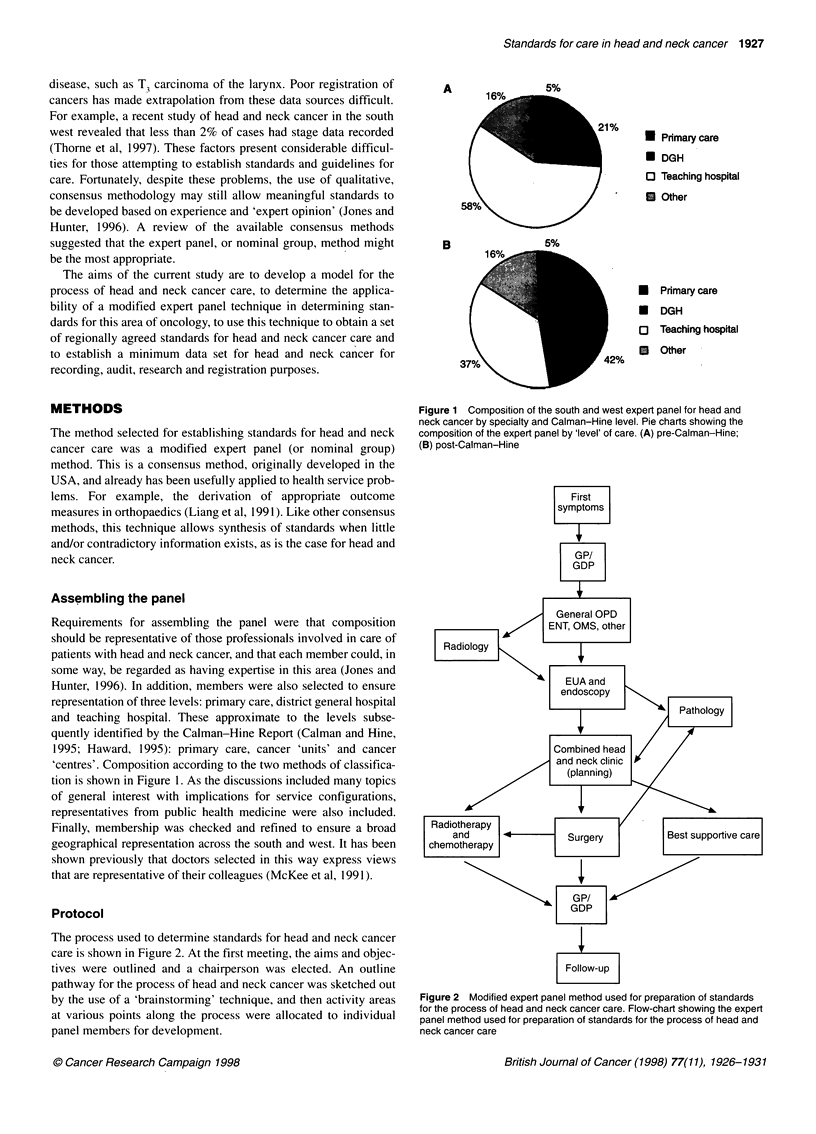

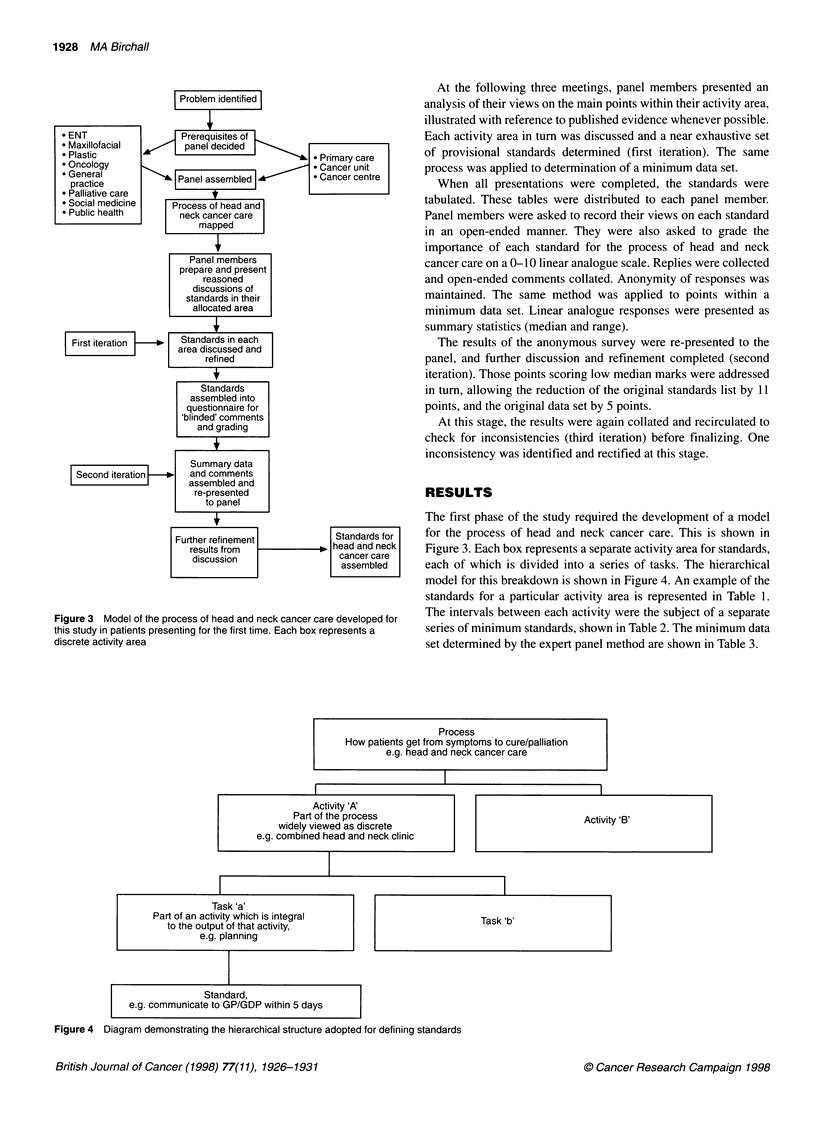

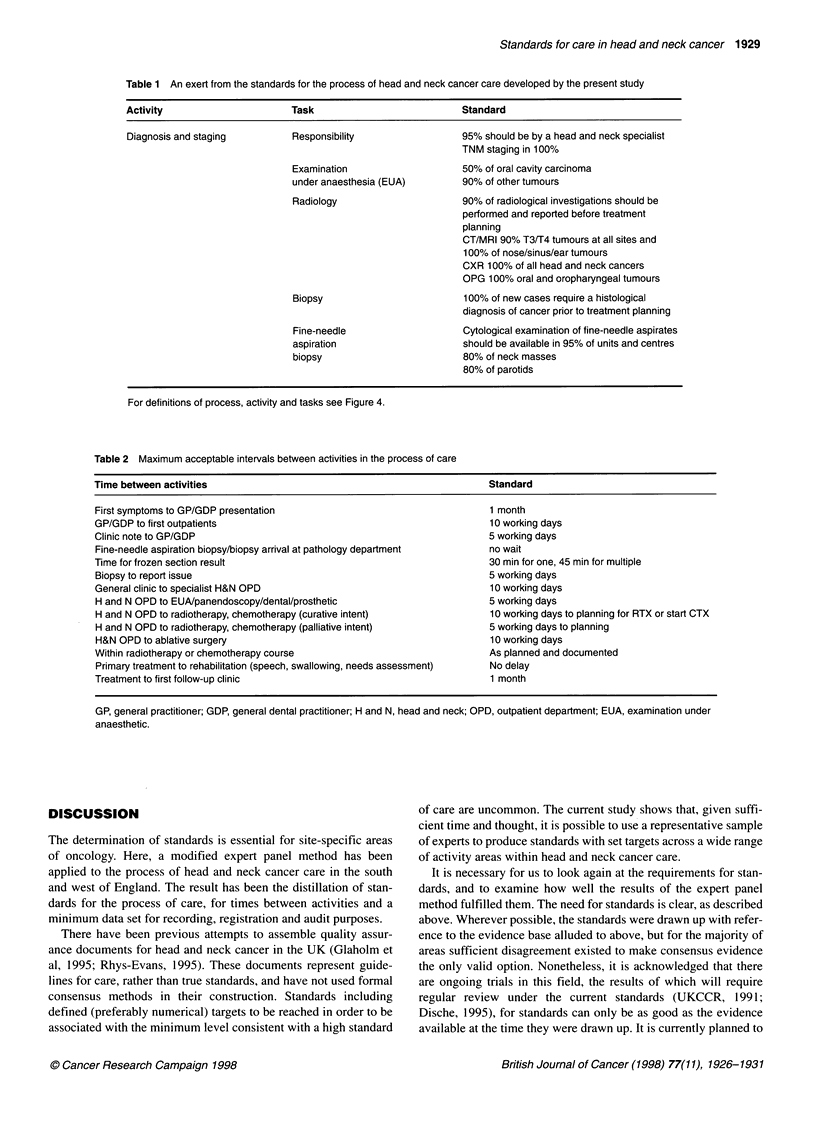

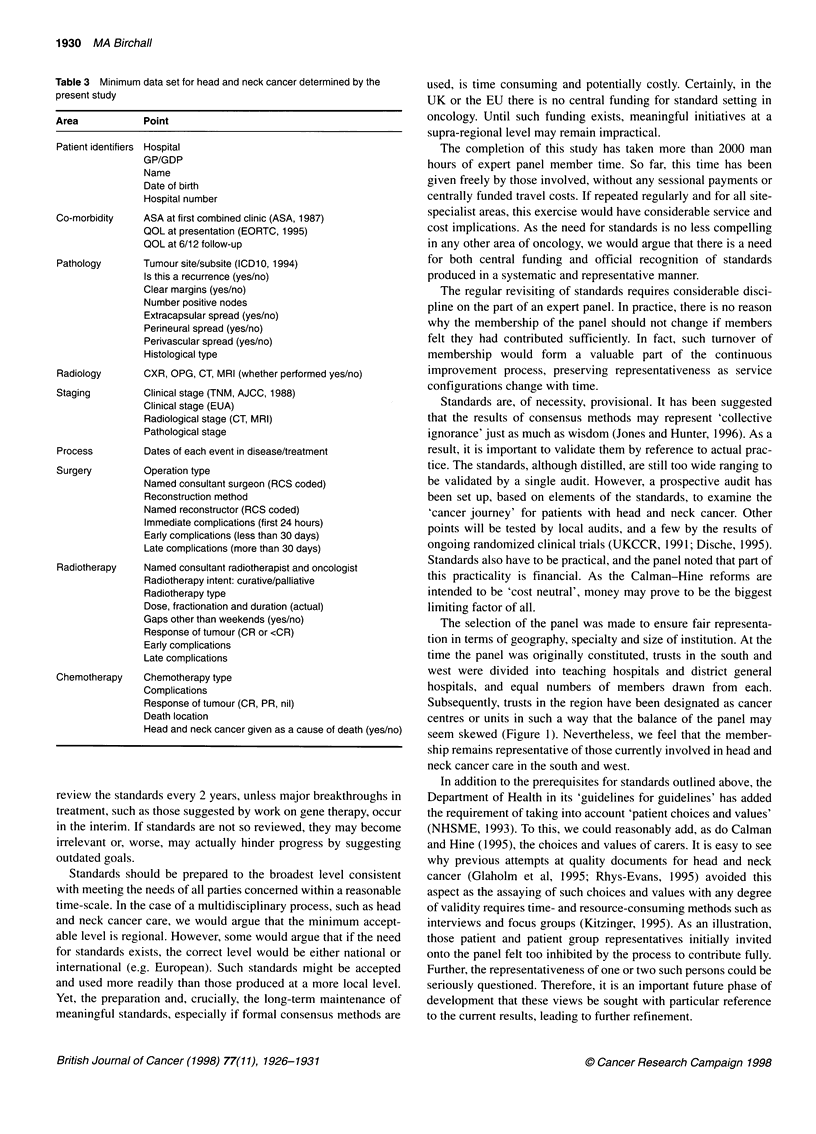

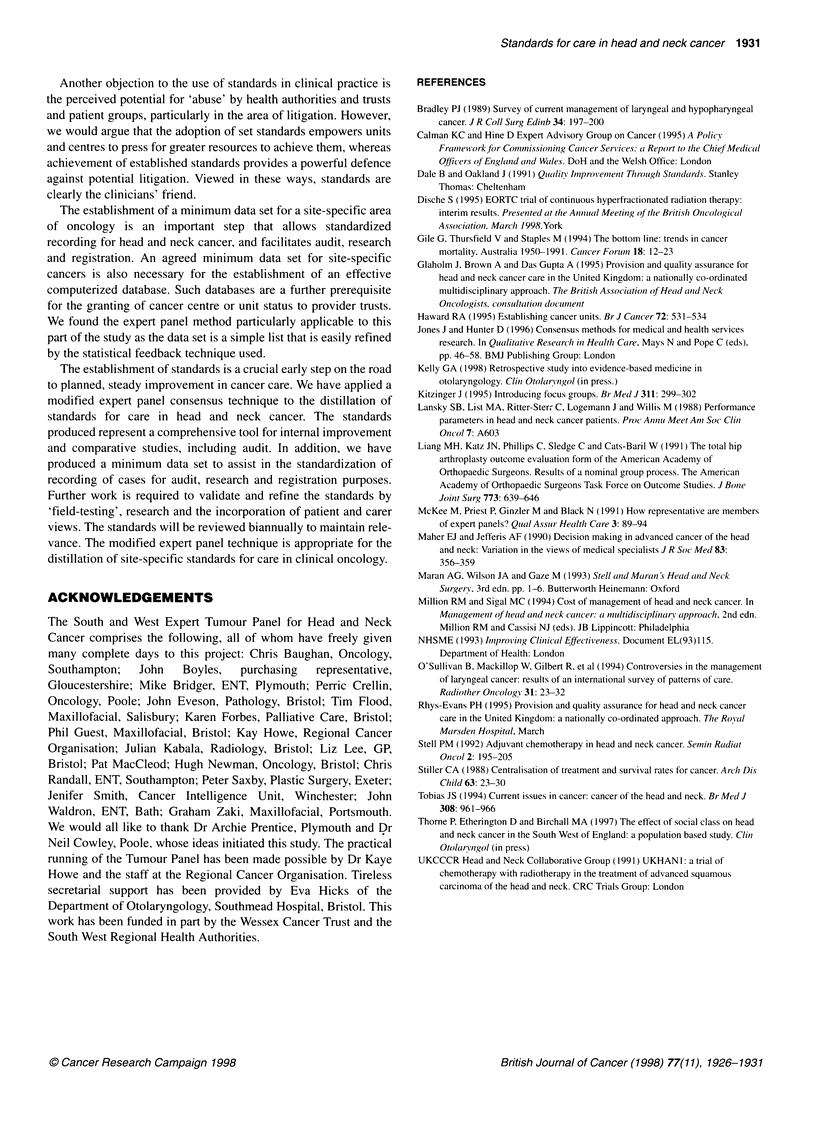


## References

[OCR_00713] Bradley P. J. (1989). Survey of current management of laryngeal and hypopharyngeal cancer.. J R Coll Surg Edinb.

[OCR_00741] Haward R. A. (1995). Establishing cancer units.. Br J Cancer.

[OCR_00752] Kitzinger J. (1995). Qualitative research. Introducing focus groups.. BMJ.

[OCR_00759] Liang M. H., Katz J. N., Phillips C., Sledge C., Cats-Baril W. (1991). The total hip arthroplasty outcome evaluation form of the American Academy of Orthopaedic Surgeons. Results of a nominal group process. The American Academy of Orthopaedic Surgeons Task Force on Outcome Studies.. J Bone Joint Surg Am.

[OCR_00772] Maher E. J., Jefferis A. F. (1990). Decision making in advanced cancer of the head and neck: variation in the views of medical specialists.. J R Soc Med.

[OCR_00770] McKee M., Priest P., Ginzler M., Black N. (1991). How representative are members of expert panels?. Qual Assur Health Care.

[OCR_00790] O'Sullivan B., Mackillop W., Gilbert R., Gaze M., Lundgren J., Atkinson C., Wynne C., Fu H. (1994). Controversies in the management of laryngeal cancer: results of an international survey of patterns of care.. Radiother Oncol.

[OCR_00800] Stell PM (1992). Adjuvant Chemotherapy in Head and Neck Cancer.. Semin Radiat Oncol.

[OCR_00804] Stiller C. A. (1988). Centralisation of treatment and survival rates for cancer.. Arch Dis Child.

[OCR_00808] Tobias J. S. (1994). Cancer of the head and neck.. BMJ.

